# Tailoring Microstructure via Rolling to Achieve Concurrent High Strength and Thermal Conductivity in Mg-Zn-Nd-Zr Alloys

**DOI:** 10.3390/ma18153578

**Published:** 2025-07-30

**Authors:** Hailong Shi, Xiaohuan Zhang, Xin Li, Yining Zhang, Siqi Li, You Wang, Xiaojun Wang, Xiaoshi Hu, Xuejian Li, Chao Xu, Weimin Gan, Chao Ding

**Affiliations:** 1 Hunan Rongtuo New Material Research Co., Ltd., Xiangtan 411201, China; hailongshi@hit.edu.cn; 2 National Key Laboratory for Precision Hot Forming, Harbin Institute of Technology, Harbin 150001, China; 24s109239@stu.hit.edu.cn (X.Z.); xinli202312@163.com (X.L.); 24s009046@stu.hit.edu.cn (Y.Z.); 24s109264@stu.hit.edu.cn (S.L.); 24s109212@stu.hit.edu.cn (Y.W.); huxiaoshi@hit.edu.cn (X.H.); 3 School of Material Science and Engineering, Harbin Institute of Technology, Harbin 150001, China; cxu@hit.edu.cn; 4 GEMS at Heinz Maier-Leibnitz Zentrum (MLZ), Helmholtz-Zentrum Hereon, Lichtenbergstr. 1, D-85748 Garching, Germany; weimin.gan@hereon.de; 5 Institute of High Energy Physics, Chinese Academy of Sciences, Beijing 100049, China; dingchao@ihep.ac.cn; 6 Spallation Neutron Source Science Center, Dongguan 523803, China

**Keywords:** Mg alloy, rolling, strength, thermal conductivity, microstructure

## Abstract

This study examined the comprehensive properties of Mg-Zn-Nd-Zr alloys in order to achieve both high strength and thermal conductivity simultaneously. The impact of rolling on the microstructure, mechanical properties, and thermal conductivity was analyzed for Mg-5Zn-xNd-0.4Zr alloys (x = 1, 2). The results indicate that the addition of Nd promotes the formation of the W phase (Mg_3_Zn_3_RE_2_), which contributes to grain boundary strengthening and enhances the overall strength. Moreover, dynamic precipitation during the rolling process leads to the formation of nanoscale MgZn_2_ and Zn_2_Zr phases, significantly improving both the strength and thermal conductivity. After rolling, both the Mg-5Zn-1Nd-0.4Zr (ZNK510) and Mg-5Zn-2Nd-0.4Zr (ZNK520) alloys exhibited a notable enhancement in thermal conductivity, with ZNK520 demonstrating superior properties due to its higher Nd content. This study highlights that optimizing alloy composition and phase evolution through rolling can markedly enhance both the mechanical and thermal properties, offering a promising strategy for the development of high-performance magnesium alloys.

## 1. Introduction

As the lightest structural metal material, magnesium (Mg) possesses a high specific strength, high specific stiffness, and excellent electrical and thermal conductivity, gaining great potential in some important fields like aerospace, high-speed transportation, and artificial intelligence robots [1,2,3,4,5]. With advancements in the semiconductor field, the trend toward higher integration is becoming increasingly pronounced. To ensure the safety and stability of components, the requirements for load-bearing structures extend beyond the mechanical properties to include performance criteria such as high thermal conductivity and lightweight characteristics [6,7,8,9,10]. Combining high strength and high thermal conductivity has long been a key direction for breakthroughs in magnesium alloy materials.

Pure magnesium has a thermal conductivity of 156 W/(m·K), ranking second only to aluminum and copper alloys among the commonly used metallic structural materials. However, it suffers from insufficient strength, which significantly limits its development [11,12,13]. The incorporation of alloying elements can enhance the CRSS (critical resolved shear stress) of the slip system in magnesium alloys, which is fundamental to the strengthening of pure magnesium materials. With an increasing concentration of alloying elements, the mechanical strengthening effect tends to exhibit an upward trend [14,15]. Nevertheless, the addition of these elements also causes a reduction in the thermal conductivity of magnesium alloys, creating a notable contradiction between strength and thermal conductivity. As the alloy content increases, the decline in thermal conductivity becomes progressively more pronounced [16]. Currently, widely used commercial magnesium alloys primarily satisfy the mechanical property requirements but fail to achieve a high thermal conductivity. For example, the thermal conductivity of AZ31 is approximately 86 W/(m·K), whereas that of AZ91 drops to 45 W/(m·K) [17]. The absence of an optimal composition ratio poses a substantial challenge to the development of magnesium alloys that possess both high strength and high thermal conductivity. Notably, when alloying elements are introduced as solid solutions, the thermal conductivity decreases by nearly an order of magnitude more when these elements are in solid solution than in a second phase [18]. Significant differences exist in the extent to which various alloying elements reduce the thermal conductivity of magnesium. Among the commonly used alloying elements, Zn exhibits the least reduction in thermal conductivity [19]. Ying et al. [20] found that in the binary Mg-Zn alloys, when the Zn content reaches 5%, the magnesium alloy can still maintain a commendable thermal conductivity of approximately 110 W/(m·K). Furthermore, the solubility of Zn in the magnesium matrix is highly temperature-dependent and decreases significantly at lower temperatures, which facilitates aging strengthening [21]. Consequently, Mg-Zn alloys are anticipated to provide mechanical strengthening while preserving good thermal conductivity. Binary Mg-Zn alloys exhibit limitations in both casting and mechanical properties, and further alloying is often needed [22]. The comprehensive properties of Mg-Zn alloys can be enhanced by adding elements such as Cu, Mn, Zr, and rare earth (RE) elements. Among the non-rare earth elements, Zr plays a key role in the grain refinement of magnesium alloys and contributes to the enhancement of their comprehensive properties. Commonly used Mg-Zn-Zr alloys, such as ZK60 and ZK20, can undergo precipitation strengthening, resulting in high strength. Li et al. [23] reported that the as-rolled Mg-2Zn-Zr alloy, after annealing and peak aging treatment, exhibited an ultimate tensile strength of 279 MPa and a thermal conductivity of 132.1 W/(m·K). In general, magnesium alloys containing RE elements tend to exhibit superior mechanical properties and reduced thermal conductivity compared with those lacking these elements [12]. However, introducing an appropriate amount of other elements into the Mg-Zn alloy can also enhance the thermal conductivity by facilitating the precipitation of a secondary phase [17,24]. Compared with heavy rare earth elements such as Gd and Y, the solid solubility of light rare earth elements like La, Ce and Nd in a magnesium matrix is lower, resulting in a relatively smaller reduction in thermal conductivity. Additionally, light rare earth elements can improve the mechanical performance by refining the grain size and modifying texture [25,26]. Zhang et al. [27] added 3% Nd into the ZK60 magnesium alloy, which enabled contributions from precipitation strengthening, grain refinement, and texture strengthening. As a result, the yield strength of the as-extruded Mg-Zn-Nd-Zr alloy increased by 77 MPa relative to the ZK60 alloy. Lv et al. [28] also found similar results. Finally, the as-extruded Mg-6Zn-1.5Nd-0.5Zr alloy obtained a yield strength as high as 408 MPa. In addition, Zhang et al. [29] and Zengin et al. [30] also investigated the effects of composition adjustment on Mg-Zn-Nd-Zr alloys, but studies focusing on thermal conductivity performance are scarce.

The development of magnesium alloys combining high strength with high thermal conductivity demands both an optimized alloying design and appropriate strengthening strategies. While commonly utilized mechanical strengthening mechanisms tend to diminish thermal conductivity, the extent of this reduction is different. Among these, solid solution strengthening results in the most significant decrease in thermal conductivity. Conversely, second-phase strengthening results in a comparatively smaller decrease in thermal conductivity. The accumulation of dislocations induces lattice distortion within the magnesium matrix, subsequently reducing the thermal conductivity [31]. Thus, the extent to which dislocation strengthening affects thermal conductivity primarily depends on the dislocation density. Fine grain strengthening is crucial during magnesium alloy design as it enhances both the strength and plasticity. Although a higher density of grain boundaries can also decrease the thermal conductivity, this reduction is generally minimal [32]. Therefore, the design of magnesium alloys that achieve a high strength and high thermal conductivity should focus on inhibiting solid solution strengthening, promoting second-phase strengthening, regulating dislocation strengthening, and enhancing grain boundary strengthening. Hot deformation is a crucial step in achieving the mechanical strengthening mechanisms and is essential for the preparation of advanced magnesium alloys. Rolling is the most effective method for producing sheets and has become one of the key processes through wrought magnesium alloy processing routes due to its ease of control. However, the limited plasticity of magnesium alloys frequently results in edge cracking during rolling, hindering the full realization of their strengthening potential. Currently, there is a scarcity of research focused on achieving high strength and high thermal conductivity magnesium alloy sheets through the rolling operation. Nevertheless, the development of such materials holds significant theoretical importance and offers broad application prospects, making it imperative to conduct in-depth studies in this area.

This work investigated the microstructural characteristics, mechanical behavior, and thermal conductivity of Mg-5Zn-xNd-0.4Zr (wt. %) alloys with x = 1 and 2. Microstructural and property modifications induced by rolling were analyzed as well as the mechanism by which Nd addition influenced the coordination between strength and thermal conductivity. This work aims to further optimize the comprehensive properties of as-rolled magnesium alloy sheets with enhanced strength and thermal conductivity and offer theoretical insights for future research.

## 2. Experimental Procedure

### 2.1. Preparation of the Mg Alloys

The alloys were prepared using the following raw materials: pure Mg, pure zinc (Zn), Mg-30Nd (wt. %), and Mg-30Zr (wt. %). Two alloy compositions were designed and prepared, as displayed in Table 1.

The melting process began by heating the resistance furnace to 760 °C. Once the furnace reached this temperature, the crucible was placed inside, and a protective gas mixture (CO_2_ + 2.4 vol. % SF6) was introduced [33,34]. When the crucible temperature reached 660 °C, the pure Mg ingot was added and allowed to fully melt. Meanwhile, the remaining materials were preheated at 250 °C. After the Mg had completely melted, the following materials were sequentially added to the melt: Mg-30Gd, Mg-30Y, and pure Zn. The materials were added in order of decreasing mass, with the heavier materials being added first. Before each material was added, the surface oxide layer of the molten metal during crucible melting was removed. The raw materials were subsequently added slowly into the melt using tongs. After each addition, the system was allowed to stabilize for about 15 min to ensure that the material had fully melted and that the elements were uniformly dispersed within the molten metal. Once all of the materials had been added, the molten alloy was maintained at 780 °C for 15 min to promote complete homogenization, followed by a stirring period of 5–10 min. The protective gas remained uninterrupted throughout the entire melting process.

During the melting process, the casting mold was preheated to 250 °C. Once the mold reached this temperature, a mold release agent was evenly sprayed inside. The mold temperature was then increased to 300 °C and maintained until the molten metal had finished settling. Before casting, the oxide layer on the surface of the molten Mg alloy was removed, and the molten alloy was immediately poured from the crucible into the preheated mold. After solidification, the cast ingot was removed from the mold.

### 2.2. Materials Characterization

The elemental composition of the as-cast Mg alloys was determined through quantitative analysis using an ICAP 7400 (Thermo Electron Corporation, Waltham, MA, USA) inductively coupled plasma-optical emission spectrometer (ICP-OES). The sample masses ranged from 10 to 100 mg.

A Zeiss Gemini 560 (Carl Zeiss AG, Oberkochen, Germany) field emission scanning electron microscope (FE-SEM) was employed to observe the microstructure of the samples. An energy dispersive spectrometer (EDS) was used to conduct a qualitative analysis of the elemental types and content in both the matrix and secondary phase. The preparation of SEM samples followed the same general requirements as the optical microscopy (OM) samples, with the decision to conduct corrosion treatment depending on the specific needs of the study. Subsequently, statistical analysis of the size, morphology, and content of the secondary phases in the SEM images was conducted using Image-Pro Plus 6.0 software.

Electron backscatter diffraction (EBSD) analysis was performed using a Zeiss Gemini 560 field emission scanning electron microscope equipped with an EBSD detector. The scanning step size was determined based on the grain size, typically set to one-tenth of the grain size to ensure sufficient and accurate data collection. EBSD samples must not only have a smooth surface, but also be free of the surface stress layer. The initial preparation method for the EBSD samples was generally the same as for the SEM samples. After mechanical polishing, it was sufficient to remove deeper scratches without completely eliminating all scratches. Subsequently, electrolytic polishing was carried out to remove the surface stress layer. The electrolyte consisted of a mixture of perchloric acid and ethanol in a 1:9 ratio. The electrolytic polishing was conducted at a voltage of 7.1 V and a current of 0.15 A for 10–15 s. After polishing, the sample was immediately removed and ultrasonically cleaned in ethanol. Liquid nitrogen was added during the polishing process to lower the electrolyte temperature and improve the resolution of the sample. AztecCrystal software (AZtec 5.1) was used for the analysis and processing of the EBSD data.

Transmission electron microscopy (TEM) was conducted using a Talos F200X microscope (FEI, Hillsboro, OR, USA). The sample preparation procedure was as follows. First, a 0.2 mm thick slice was cut using electrical discharge wire cutting. This slice was then polished on SiC sandpaper until the thickness was reduced to below 50 μm. Finally, a Φ3 mm disc was thinned using a Gatan 695 ion thinning instrument (Gatan, Pleasanton, CA, USA). Introduction of the ion milling instrument and parameters. Ion gun: A Penning-type ion gun equipped with miniature magnets, designed for focused ion beam operation, with no consumable parts. Milling angle: Adjustable from +10° to −10°, each ion gun can be independently controlled. Sample stage rotation: The sample stage is rotatable, with an adjustable rotation speed ranging from 1 to 6 rpm. Sample stage movement range: The stage allows movement in both the X and Y directions, with a range of ±0.5 mm. Ion beam energy: Ranges from 0.1 keV to 8 keV, with automatic optimization of the ion beam current at different voltages. Vacuum system: Oil-free mechanical pump combined with a molecular pump system. The transmission electron microscope was equipped with an energy dispersive spectrometer (EDS), which was used to perform compositional analysis of the samples.

Tensile tests at room temperature were carried out on an Instron 5569 universal testing machine (Instron, Boston, MA, USA)and strain data were recorded using an extensometer. Tensile tests were conducted along the rolling direction (RD) of the plate samples, and the tensile rate was set to 0.5 mm/min. The samples, cut by wire electrical discharge machining, were polished on both the surface and sides with #1000 sandpaper to eliminate any macro-defects that might have developed during processing, which could influence the accuracy of the test results. After preparation, the actual gauge length was measured precisely using a caliper.

Thermal conductivity was measured using an LFA467 laser flash analyzer (NETZSCH, SELB, Germany). The samples were circular discs with a diameter of 12.7 mm and a thickness of 1.0–2.5 mm. The testing direction was perpendicular to the plane of the plate (normal direction, ND). The sample surfaces were carefully polished using sandpaper to eliminate surface contaminants and oxide layers. A micrometer was employed to measure the actual thickness of the samples, and the density was calculated using the Archimedes method. Before testing, both surfaces of the samples were uniformly coated with carbon spray.

The rolling experiments were conducted using a conventional two-roll cold rolling mill. The initial samples, measuring 45 mm × 30 mm × 8 mm, were pre-machined with a chamfer on one of the short edges to facilitate subsequent rolling. Throughout the process, the rollers were not heated, and a constant rolling speed of 20 m/min was maintained. The samples were heated and held in a box-type resistance furnace. A multi-pass hot rolling process was employed in this study. Before the first pass, the samples were held at the preset rolling temperature for 30 min. After each rolling pass, the samples were quickly returned to the resistance furnace for a 10-min holding period to reheat them to the preset temperature and relieve partial deformation, ensuring the continuity of the rolling process. After the final pass, the samples were water-quenched to preserve the microstructure in its current state.

The thermal conductivity and tensile data of all samples were measured three times and averaged to ensure accuracy.

## 3. Results and Discussion

### 3.1. Microstructure of Mg-5Zn-xNd-0.4Zr Alloys

**Figure 1:**
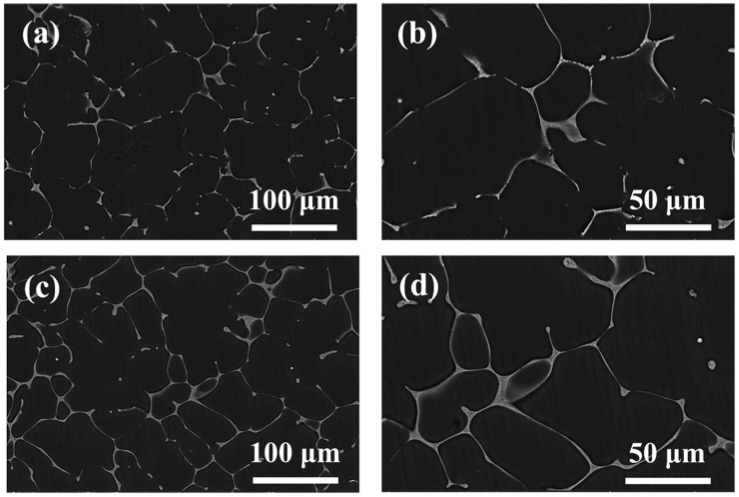
SEM-SE images of the as-cast (**a**,**b**) ZNK510 and (**c**,**d**) ZNK520 samples.

**Figure 2:**
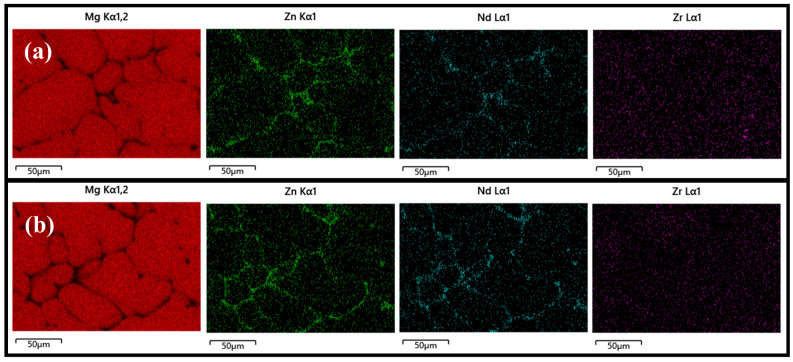
Elemental mapping of the as-cast (**a**) ZNK510 and (**b**) ZNK520 samples.

Similar to the analytical approach used by Park et al. [35], we specifically analyzed the impact of phase transformations on the material properties. Figure 1 displays the SEM-SE (scanning electron microscope-secondary electron) micrographs of the as-cast Mg–5Zn-xNd-0.4Zr alloys. Besides the α-Mg phase, both samples contained a network eutectic phase. An increase in Nd content led to a rise in the area fraction of the network second phase, from 2.90% in ZNK510 to 4.61% in ZNK520. In terms of distribution, the second phase transitioned from a semi-continuous network structure to a more continuous network structure. Figure 2 illustrates the elemental distribution of the two as-cast Mg-5Zn-xNd-0.4Zr samples based on energy dispersive spectroscopy (EDS) surface scanning. The network-structured second phase was identified as consisting of Mg, Zn, and Nd, implying that Nd addition regulates the formation process and drives the transformation of Zn from a solid solution into a second-phase compound. Furthermore, Zr was homogeneously distributed within the matrix and did not contribute to the formation of micron-scale secondary phases.

**Figure 3:**
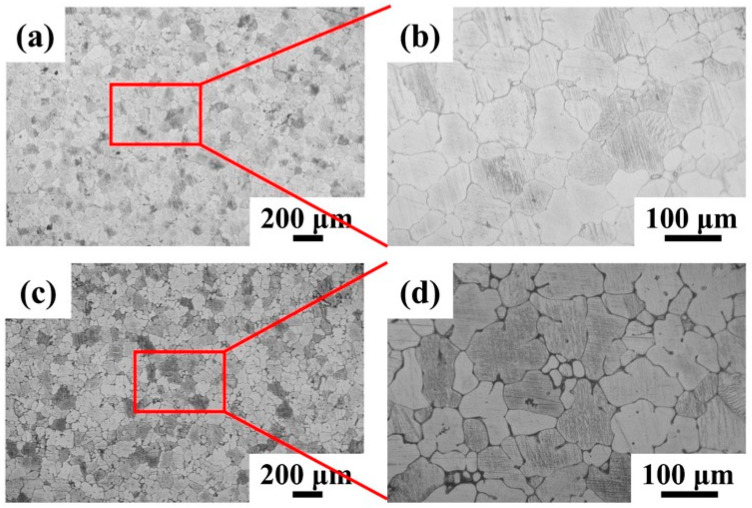
OM images of the as-cast (**a**,**b**) ZNK510 and (**c**,**d**) ZNK520 samples.

Figure 3 presents the OM images of the two as-cast Mg-5Zn-xNd-0.4Zr samples, where numerous well-defined grain boundaries were clearly visible. This observation suggests that the addition of Nd and Zr effectively contributed to grain refinement. Furthermore, the as-cast ZNK520 sample, which contained a higher concentration of Nd, demonstrated a reduced grain size, as illustrated in Figure 3c,d. This phenomenon may be attributed to the enrichment of additional Nd at the solid/liquid interface behavior in the course of solidification, which leads to a greater degree of undercooling occurring within the solute diffusion layer. This undercooling reduces the atomic diffusion rate, thereby increasing the nucleation rate of the grains [36]. Additionally, the second phase was mainly concentrated at the grain boundaries, further restricting grain boundary migration. A similar behavior has been reported in other magnesium alloys incorporating light rare earth elements [37].

**Figure 4:**
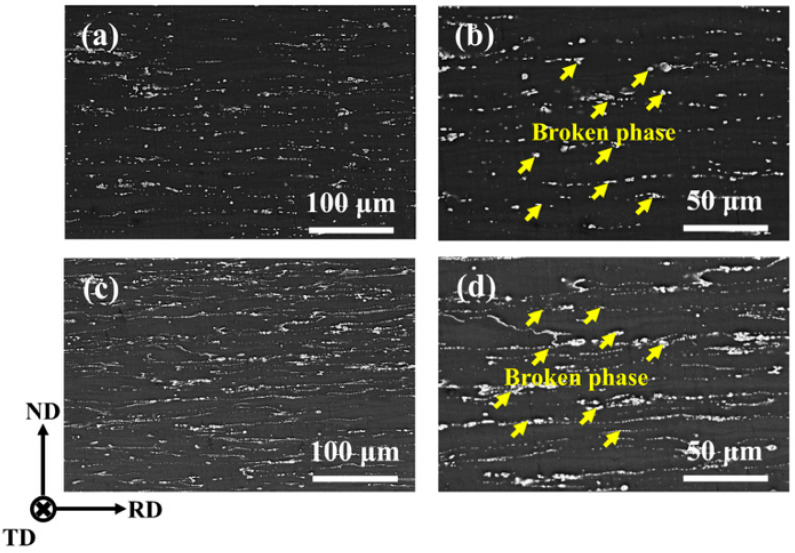
SEM-SE images observed on the RD-ND plane of the as-rolled (**a**,**b**) ZNK510 and (**c**,**d**) ZNK520 samples.

The as-cast Mg-5Zn-xNd-0.4Zr samples underwent multi-pass rolling at 450 °C following a homogenization treatment, resulting in a total thickness reduction of 87.5%. Figure 4 presents the SEM-SE images of the as-rolled ZNK510 and ZNK520 samples in the RD (rolling direction)-ND (normal direction) plane; the second phase was distributed along the RD. Morphologically, the network eutectic phase of the as-cast samples was broken during rolling and transformed into spherical particles, as marked by the yellow arrows in Figure 4b,d. Table 2 lists the area fraction and average size of the second phase in the two as-rolled Mg-5Zn-xNd-0.4Zr samples. These results demonstrated that the area fraction of the second phase in the as-rolled ZNK510 and ZNK520 samples was 4.50% and 8.03%, respectively, which represents a significant increase relative to the as-cast samples. The ZNK520 sample with a higher Nd content always exhibited a higher fraction of the second phase during the preparation process. In terms of the second-phase size, the as-rolled ZNK520 sample demonstrated a more pronounced degree of fragmentation, resulting in a higher number of smaller particles. Eventually, the as-rolled ZNK520 sample showed a smaller average size. Additionally, a significant presence of submicron second phases was observed in the as-rolled samples, which may have been due to the breaking of the initial network second phase or the dynamic precipitation occurring during the rolling process.

**Figure 5:**
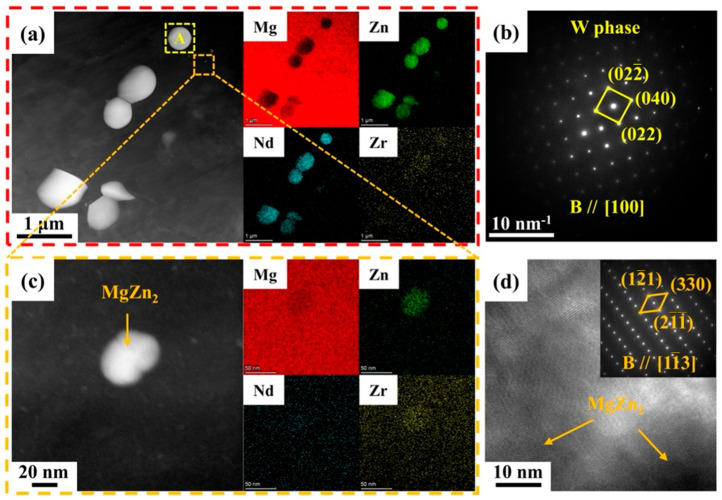
(**a**) HAADF images and elemental distribution of the W phase, (**b**) SAED images of the W phase, (**c**) HAADF images and the corresponding elemental distribution maps of the MgZn_2_ phase at high magnification, and (**d**) HRTEM and corresponding FFT images of the MgZn_2_ phase of the as-rolled ZNK520 sample.

**Figure 6:**
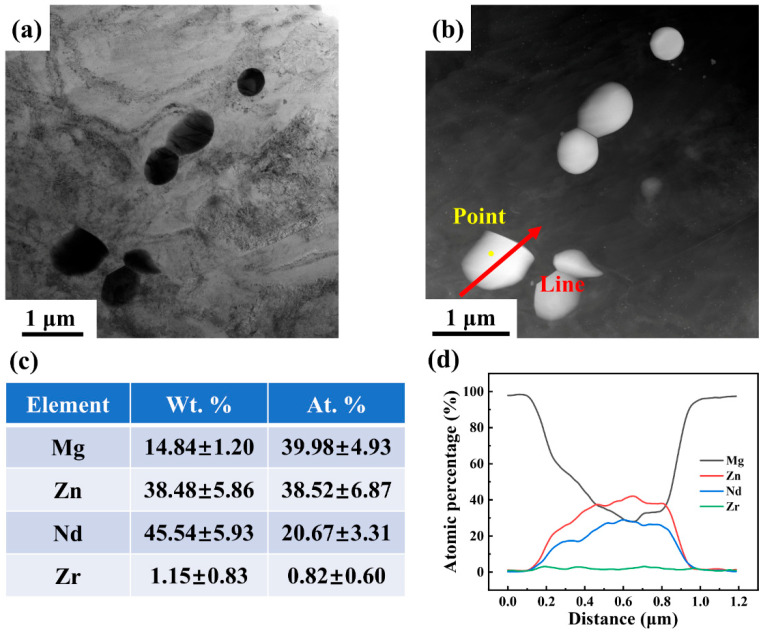
(**a**) TEM bright field image, (**b**) HAADF image and corresponding EDS (**c**) point, and (**d**) line analyses of the as-rolled ZNK520 sample.

To further investigate the evolution of the second phase in the Mg-5Zn-xNd-0.4Zr samples, TEM was used to characterize the as-rolled ZNK520 sample. As illustrated in Figure 5a, a substantial number of submicron second phases could be observed at low magnification, and the enrichment of Zn and Nd elements in the phase composition aligned with the characteristics of the network second phase observed in the as-cast sample. According to point and line scanning on the submicron second phase, it can be inferred that this was the W phase (Mg_3_Zn_3_RE_2_), as depicted in Figure 6. The atomic percentages of Mg and Zn were comparable, and was 1.5 times that of the Nd content.

Based on the selective area electron diffraction (SAED) analysis of particle A, located in the yellow box in Figure 5a, it can be seen that the Mg-Zn-Nd phase in this study corresponded to the W phase, which is consistent with previous reports identifying it as the main second phase in other Mg–Zn–RE alloys [28,38,39]. The morphology of the second phase, extending from the lower left to the upper right in Figure 5a, revealed that the submicron W phase comprised both particles, exhibiting distinct angular characteristics and spherical particles of varying sizes. This observation indicates that the W phases with these two morphologies originated from the fragmentation of the initial network phase and dynamic precipitation during the rolling process, respectively. In addition to the submicron W phase, finer nano-sized precipitates were also observed, as indicated in the orange box in Figure 5a. Figure 5c presents the high-angle annular dark field (HAADF) image and elemental distribution of the nano-sized precipitates at high magnification. The composition primarily consisted of Zn, with negligible amounts of Nd. High-resolution transmission electron microscopy (HRTEM) images and the corresponding fast Fourier transform (FFT) pattern analyses confirmed that the precipitated phase was the MgZn_2_ phase, as illustrated in Figure 5d.

**Figure 7:**
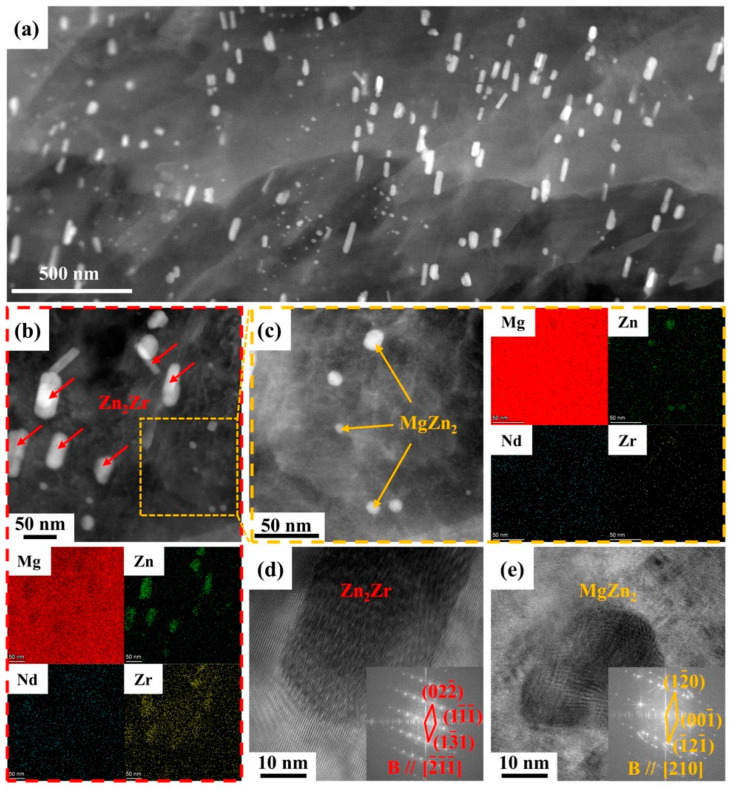
(**a**) HAADF images of the dynamic precipitated phases at low magnification, (**b**) HAADF images and element distribution of (**b**) rod-like and (**c**) spherical precipitates at high magnification, and HRTEM images and corresponding FFT images of the (**d**) Zn_2_Zr and (**e**) MgZn_2_ phases in the as-rolled ZNK520 sample.

In addition, numerous nano-sized precipitates were observed in other regions of the as-rolled ZNK520 sample, and the morphology mainly included short rod-like and spherical, as illustrated in Figure 7a. The elemental distribution analysis revealed that these nano-sized precipitates lacked the Nd element. Specifically, the short rod-like phase was composed of Zn and Zr, whereas the spherical phase contained only Zn, as depicted in Figure 7b,c. The HRTEM and FFT results confirmed that the short rod-like phase matched the Zn_2_Zr phase, while the spherical phase was identified as the MgZn_2_ phase, as shown in Figure 7d,e.

In this study, the light rare earth element Nd was introduced to form the W phase through its combination with Zn. Although it was accompanied by dynamic precipitation behavior, it mainly existed in the form of submicron size during the rolling process. Consequently, the W phase exhibited a limited strengthening effect and enhanced mechanical strength mainly via indirect pathways. In contrast, Zn served as the dominant alloying addition in this composition, contributing significantly to the precipitation of nanoscale phases during rolling. The abundance of nano-sized precipitates in the ZNK520 sample will contribute significantly to the improvement in the strength, and this precipitation behavior will also serve a function in improving the thermal conductivity. Additionally, Zr, as an extra alloying element, not only influences the thermal conductivity, but also alters the formation of the precipitated phase, thereby affecting the strength.

**Figure 8:**
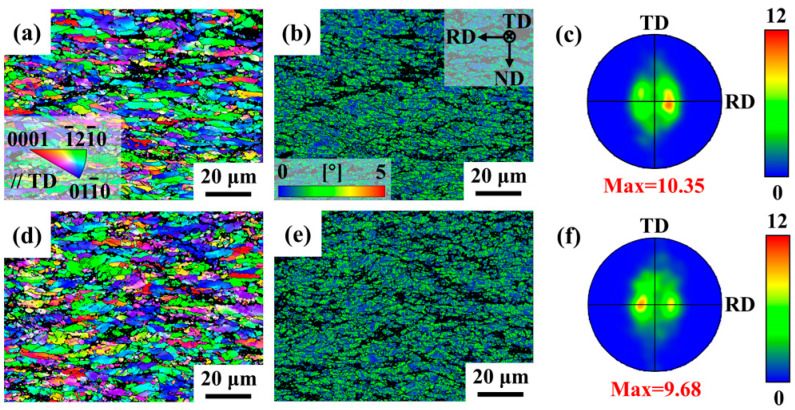
EBSD results including: (**a**,**d**) inverse pole figure (IPF) maps, (**b**,**e**) kernel average misorientation (KAM) maps, and (**c**,**f**) (0001) pole figure (PF) maps of the (**a**–**c**) as-rolled ZNK510 and (**d**,**e**) ZNK520 samples.

**Figure 9:**
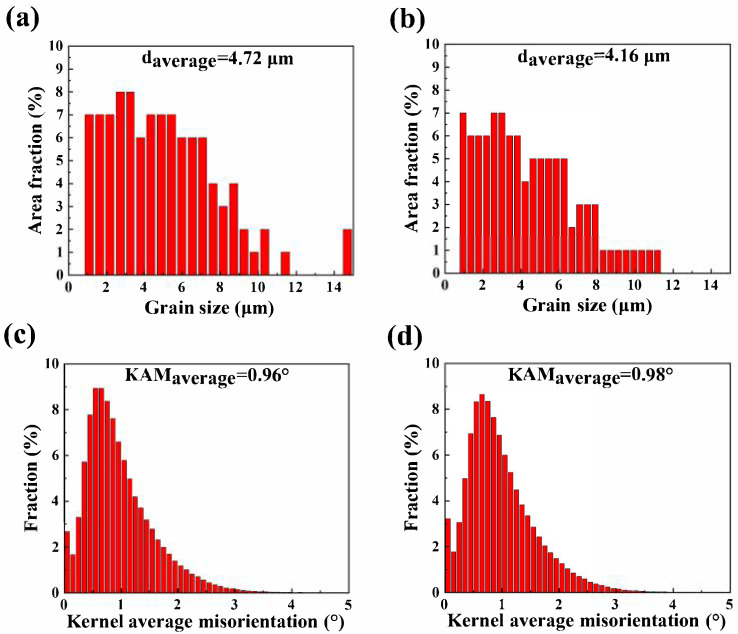
(**a**,**b**) Grain size and (**c**,**d**) KAM distribution histogram of the as-rolled (**a**,**c**) ZNK510 and (**b**,**d**) ZNK520 samples.

Figure 8 presents the EBSD results for the as-rolled ZNK510 and ZNK520 samples. The black region in the figure represents the unanalyzed portion of the second phase and its surrounding area. The rolling of the final pass with a large thickness reduction promoted the elongation of the grains along the RD and the compression along the ND, resulting in substantial distortion, as illustrated in Figure 8a,b,d,e. Color gradients in the inverse pole figure (IPF) maps serve as indicators of misorientation developed during plastic deformation, which may contain subgrains and a considerable number of dislocations. The grain size distribution and kernel average misorientation (KAM) distribution for both samples are shown in Figure 9. The average grain sizes for the as-rolled ZNK510 and ZNK520 samples were 4.72 μm and 4.16 μm, respectively, while the two samples exhibited average KAM values of 0.96° and 0.98°, respectively.

As a hard second phase, the W phase is difficult to deform during the rolling process, and can only be broken and partially dissolved. In the magnesium matrix, dislocations are prone to accumulate at the interface with the W phase. Furthermore, the average size of the W phase in Mg-5Zn-xNd-0.4Zr samples typically exceeds 1 μm. As a result of the synergistic effect of a high dislocation density and the particle-stimulated nucleation (PSN) mechanism, the Mg-5Zn-xNd-0.4Zr samples gradually refine grains and modify texture through dynamic recrystallization (DRX) during hot deformation and static recrystallization (SRX) during reheating between passes [40,41,42].

The as-rolled ZNK520 sample exhibited a higher fraction of secondary phase, which resulted in the accumulation of more dislocations during the rolling process and provided a stronger driving force for grain size refinement. In terms of orientation, both the as-rolled ZNK510 and ZNK520 samples displayed basal texture with polar axis splitting along the RD. ZNK520 exhibited a maximum texture intensity of 9.68, which was slightly lower than the 10.35 recorded for ZNK510. The observed weakening of texture intensity may be attributed to two factors: (1) a significant amount of W influences the deformation mode of the surrounding matrix during hot deformation, and (2) the presence of more W phase enables the ZNK520 sample to undergo more significant recrystallization behavior during rolling.

Magnesium alloy sheets have strong heat exchange capabilities during the rolling process, especially when the rolls lack heating ability, leading to a significant temperature drop at the moment the sheet comes into contact with the rolls. Therefore, the final rolled samples in this study were mainly composed of deformed grains, with the dynamic recrystallization behavior being suppressed. Moreover, the nanoscale precipitates formed during the rolling process primarily serve to strengthen the grain boundaries, rather than promoting dynamic recrystallization [25]. Grain refinement and texture evolution during multi-pass rolling jointly contributed to the optimization of the final microstructure. Among these processes, recrystallization behavior plays an essential role. As depicted in Figure 8 and Figure 9, the grain size and shape of the final rolled sample were relatively uniform. It can be inferred that the microstructure of the sample before deformation must be relatively uniform. Therefore, the insulation process between rolling passes facilitates the uniformization of the microstructure through static recrystallization behavior, promoting the continuous and stable progression of the entire rolling process.

### 3.2. Mechanical Properties of Mg-5Zn-xNd-0.4Zr Alloys

**Figure 10:**
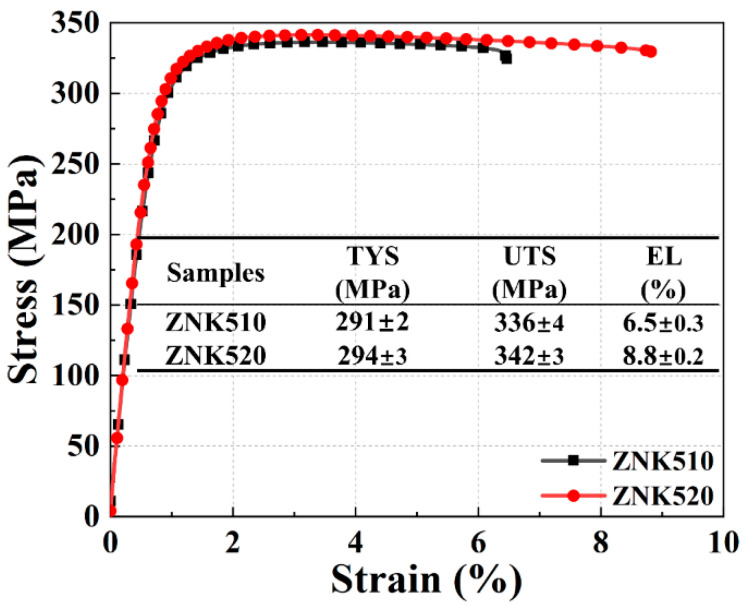
Tensile stress–strain curves of the as-rolled ZNK510 and ZNK520 samples.

The room temperature tensile curves of the ZNK510 and ZNK520 samples are shown in Figure 10, which also includes a summary of their mechanical properties. After rolling, significant enhancement in the mechanical properties of the Mg-Zn-Nd-Zr alloy was achieved. For the rolled ZNK510 sample, the yield strength was 291 MPa, the tensile strength was 336 MPa, and the elongation was 6.5%. The rolled ZNK520 sample slightly outperformed the ZNK510 sample in terms of mechanical properties, with a yield strength of 294 MPa, tensile strength of 342 MPa, and elongation of 8.8%.

Grain boundary strengthening is fundamental to improving the strength of Mg alloys, according to the Hall–Petch equation [43]:

(1) $${∆\sigma }_{GB}=Kd^{-\frac{1}{2}},$$

where ${∆\sigma }_{GB}$ represents the role of the grain boundary to the yield strength, K is the Hall–Petch slope, also known as the stress concentration factor (K = 272 MPa μm^1/2^ [44]), and d is the average grain size. The grain boundary strengthening contribution to the ZNK510 and ZNK520 samples was approximately 125 MPa and 133 MPa, respectively.

In this study, dislocation strengthening further led to the improved strength of the Mg alloy, and the resulting increase in strength due to dislocations can be determined using the following equation [45]:

(2) $${∆\sigma }_{dis}=\alpha Gb\sqrt{\rho },$$

where ${∆\sigma }_{dis}$ represents the role of dislocation strengthening to the yield strength, α is a constant, G is the shear modulus, b is the Burgers vector, and ρ is the dislocation density. The contribution of dislocation strengthening to the ZNK510 and ZNK520 samples was approximately 102 MPa and 103 MPa, respectively. An increase in dislocation density enhanced the yield strength of the alloy; however, this improvement was accompanied by a reduction in plasticity and work hardening capacity.

The impact of the second phase on the alloy’s yield strength can be calculated through the following equation [46,47]:

(3) $${∆\sigma }_{p}=\frac{Gb}{2\pi \lambda \sqrt{1-\nu }}ln\frac{d}{b},$$

where ${∆\sigma }_{p}$ represents the contribution of the precipitate phase to the yield strength, G is the shear modulus of Mg, b is the Burgers vector, λ is the average distance between adjacent phases, ν is the Poisson’s ratio of Mg, and d is the average diameter of second phase. For the W phase in this work, its micron-scale size contributes relatively little to the strength. However, during the rolling process, the higher content of the W phase in the ZNK520 sample enhanced the contributions from grain refinement strengthening and dislocation strengthening, playing an indirect strengthening role. Additionally, the dynamically precipitated nanoscale MgZn_2_ and Zn_2_Zr phases during the rolling process contributed significantly to the strength of the ZNK520 sample through dispersion strengthening. Ultimately, both rolled Mg-Zn-Nd-Zr alloys exhibited high strength.

In this study, to achieve higher thermal conductivity, the influence of solid solution strengthening on the strength of the Mg-Zn-Nd-Zr alloy was insignificant. Based on the above analysis, the distinction in strength between the ZNK510 and ZNK520 alloys mainly originated from grain boundary strengthening. However, experimental results showed that the difference in yield strength between the two alloys was smaller than expected. This may be due to the higher content of Zn atoms in the solid solution of the cast ZNK510 alloy, which allowed for the precipitation of more nanoscale strengthening phases during the rolling process, partially compensating for the deficiency in grain boundary strengthening.

In terms of plasticity, the rolled ZNK520 sample was slightly superior to the ZNK510 sample. This is primarily because the fine grain size and relatively weakened texture helped enhance the alloy’s ability to undergo coordinated deformation, ultimately maintaining good plasticity. In small-sized grains, dislocation slip distances are shorter and local stress concentration is less severe, allowing for more uniform overall deformation of the alloy. During tensile testing along the rolling direction (RD), the basal planes of some grains may rotate at a certain angle along the RD, changing from a hard orientation to a soft orientation. The weaker the basal plane texture in the sample, the more favorable it is for initiating basal slip and exhibiting stronger coordinated deformation capability. Moreover, although the ZNK520 sample contained a large amount of the secondary phase, after rolling, the secondary phase was divided into fine spherical particles and uniformly distributed within the matrix, which did not significantly harm the material’s plasticity.

### 3.3. Thermal Conductivity of Mg-5Zn-xNd-0.4Zr Alloys

**Figure 11:**
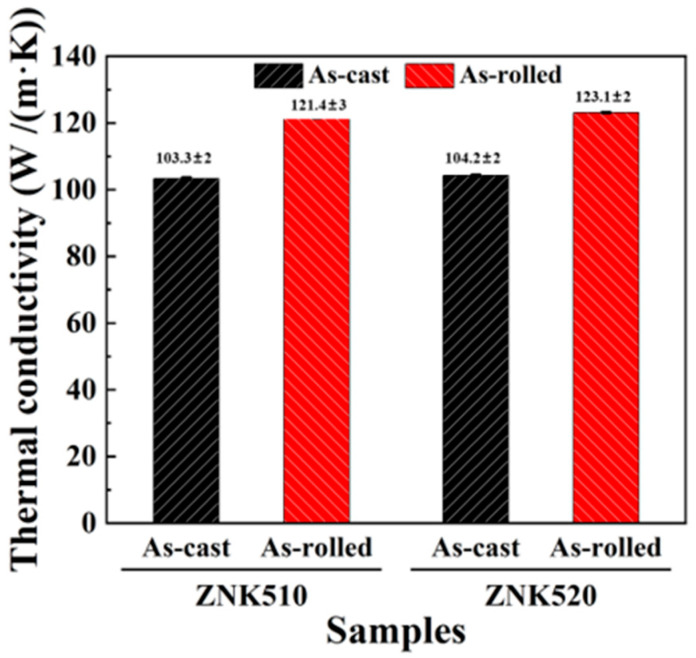
Thermal conductivity of the as-cast and as-rolled Mg-5Zn-xNd-0.4Zr samples.

The room temperature thermal conductivity of the two Mg-Zn-Nd-Zr alloys in both cast and rolled states is shown in Figure 11. The thermal conductivity at room temperature of the ZNK510 and ZNK520 samples in the cast state was 103.3 W/(m∙K) and 104.2 W/(m∙K), respectively. However, after rolling, the ambient temperature thermal conductivity of the ZNK510 and ZNK520 samples increased to 121.4 W/(m∙K) and 123.1 W/(m∙K), respectively. In the same state, the ZNK520 sample, with a higher Nd content, exhibited a higher thermal conductivity. Both alloys had Zn as the main alloying element, with only differences in the Nd content. In both alloys, Nd was mainly present as the W phase, with a higher Nd concentration in the ZNK520 alloy leading to a greater formation of the W phase, which diminished the Zn atom content in the matrix’s solid solution, causing higher thermal conductivity.

It is worth noting that due to the dynamic precipitation of the second phase during the rolling process, both alloys exhibited a substantial increase in ambient temperature thermal conductivity and thermal diffusivity, relative to the initial cast samples in the rolled state. Nandihalli et al. [48] found that the heat transfer in the material or alloy depends on the acoustic mismatch (difference in sound velocity) and differences in the physical and chemical properties at the interfaces of different constituents. The results of this study also confirm that the micron-scale primary phases and nanoscale dynamic precipitates in this work serve a beneficial function in enhancing the high strength and thermal conductivity of the rolled Mg alloys.

As seen in Figure 8, both the rolled ZNK510 and ZNK520 samples stored a large number of dislocations, which will inevitably cause the scattering of electron migration, causing in a significant reduction in thermal conductivity [49]. Additionally, the finer grain size also exerted some inhibitory effects on the thermal conductivity of the magnesium alloy. However, the results show that after rolling, the thermal conductivity of both Mg-Zn-Nd-Zr samples significantly improved compared with the cast state samples, which is an effect not achieved in many previous studies [50,51]. This phenomenon is mainly due to the substantial transformation of alloying components from a solid solution form to the second-phase form during the rolling process. This transformation significantly enhances the thermal conductivity, compensating for the inhibiting effects of other factors and promoting the strengthening impact on the mechanical properties of the magnesium alloy.

For sheet materials, the thermal conductivity in the ND direction was primarily of interest, and the basal texture formed in the rolled samples was a key contributor to the improvement in thermal conductivity. For magnesium with an HCP structure, the atomic separation along the c-axis exhibits a relatively large value, and when the electrons and phonons move along the c-axis, the resistance they encounter is small. Therefore, for the rolled magnesium alloy sheets, the ND direction exhibited relatively good thermal conductivity [52].

**Figure 12:**
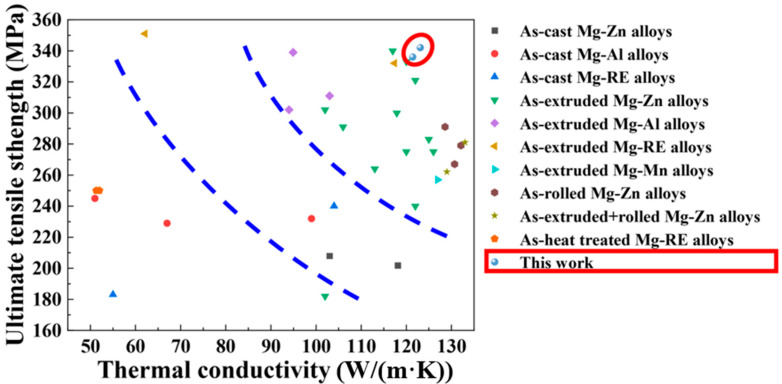
Ultimate tensile strength versus the thermal conductivity of some previous magnesium alloys [7,12,16,23,25,31,52,53,54,55,56,57,58] and the Mg-5Zn-xNd-0.4Zr alloys in this work.

The strength characteristics and thermal conductivity of magnesium alloys present an inherently conflicting mechanism, as enhancing one property often compromises the other. This is true in many other composites [48]. Achieving high strength and thermal conductivity simultaneously requires promoting the improvement in one property while minimizing the detrimental effects on the other. Figure 12 summarizes the previous studies on the tensile strength and thermal conductivity of magnesium alloys. The rolled Mg-Zn-Nd-Zr alloy in this study demonstrated excellent strength-thermal performance. Additionally, the above results indicate that regulating the thermal conductivity of magnesium alloys through compositional design should not be limited to the selection of alloying element types and contents. By combining the alloy composition design with thermal deformation processes, adjusting the phase state of alloying elements may hold the potential to fully enhance the mechanical properties while maintaining high thermal conductivity, and could offer a novel method for designing magnesium alloys with high strength and thermal conductivity.

## 4. Conclusions

This study explored the refinement of Mg-Zn-Nd-Zr alloys with optimized strength and thermal conductivity. The impact of alloy composition, phase states, and rolling processes on the microstructure, mechanical properties, and thermal conductivity was thoroughly analyzed. The main conclusions were drawn as follows:

1. The rolled Mg-Zn-Nd-Zr alloys exhibited a combination of elevated strength and thermal conductivity, with ZNK520 showing the best overall performance due to its higher Nd content and increased second-phase formation.
2. Grain boundary strengthening, the dynamic precipitation of nanoscale phases during rolling, and microstructure evolution, particularly the formation of basal textures in the ND direction, significantly enhanced both the mechanical properties and thermal conductivity of the alloy.
3. Combining composition design with thermal deformation processes, including phase state regulation, is an effective strategy in the pursuit of Mg alloys with simultaneous high strength and thermal conductivity.

## Figures and Tables

**Table 1:** Chemical composition of the as-cast Mg-5Zn-xNd-0.4Zr alloys (wt. %).

Alloys	Nominal Composition	Actual Composition			
		Mg	Zn	Nd	Zr
ZNK510	Mg-5Zn-1Nd-0.4Zr	92.88 ± 2.34	5.56 ± 0.97	1.02 ± 0.03	0.54 ± 0.0
ZNK520	Mg-5Zn-2Nd-0.4Zr	91.55 ± 3.12	5.88 ± 0.88	2.18 ± 0.06	0.39 ± 0.1

**Table 2:** The statistical results of the second phase in the as-rolled ZNK510 and ZNK520 samples.

Samples	Area Fraction (%)	Average Size (μm)
As-rolled ZNK510	4.50	2.36
As-rolled ZNK520	8.03	2.23

## Data Availability

The original contributions presented in this study are included in the article. Further inquiries can be directed to the corresponding author(s).
